# RANKL inhibition improves muscle strength and insulin sensitivity and restores bone mass

**DOI:** 10.1172/JCI169317

**Published:** 2023-02-15

**Authors:** Nicolas Bonnet, Lucie Bourgoin, Emmanuel Biver, Eleni Douni, Serge Ferrari

Original citation: *J Clin Invest*. 2019;129(8):3214–3223. https://doi.org/10.1172/JCI125915

Citation for this corrigendum: *J Clin Invest*. 2023;133(4):169317. https://doi.org/10.1172/JCI169317

The authors recently became aware that an incorrect image was shown in Figure 6F for the *Pparb^–/–^* Opg-Fc sample. The correct figure is shown below.

The authors regret the error.

## Figures and Tables

**Figure 6:**
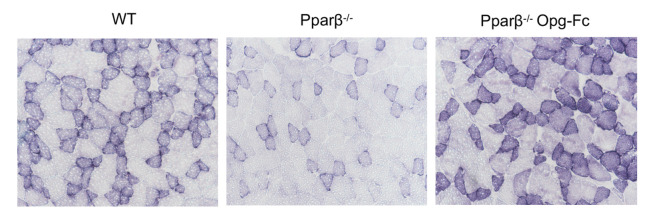
Bone, muscle, and glucose phenotype of *Pparb^–/–^* mice treated by OPG-Fc. (**A** and **B**) Skeletal muscle volume of the limb and fat infiltration in muscle evaluated by in vivo microCT. (**C**) Maximal speed evaluated on treadmill normalized by gastrocnemius mass. (**D**) Limb force evaluated by handgrip normalized by gastrocnemius mass (*n* = 8 per group). (**E**) Body temperature evaluated by infrared camera (*n* = 8 per group). (**F**) Muscle fiber type, number, and area. Note the type I fibers in blue dark and type II fibers in light blue. Original magnification is ×10. (**G**) ITT AUC (*n* = 8 per group). (**H**) GTT. (**I**) Relative protein expression in the gastrocnemius. Hatch marks correspond to mice that have received an acute injection of insulin. (**J**) Relative mRNA expression of insulin signaling in soleus. (**K**) Relative mRNA expression of *Nfkb* signaling in soleus (*n* = 6 per group). Statistical differences were assessed by 1-way ANOVA. ^†^*P* < 0.05, ^‡^*P* < 0.01 significant difference versus WT. Bars show mean ± SEM.

## Version 1 02/15/2023

Electronic publication

